# Predictive factors of late cholangitis in patients undergoing pancreaticoduodenectomy

**DOI:** 10.1186/s12957-017-1301-6

**Published:** 2018-01-31

**Authors:** Yasuhiro Ito, Yuta Abe, Minoru Kitago, Osamu Itano, Yuko Kitagawa

**Affiliations:** 1 Department of Surgery, Saiseikai Yokohamashi Tobu Hospital, Yokohama, Kanagawa 230-0012 Japan; 2 Department of Surgery, School of Medicine, Keio University, 35 Shinanomachi, Shinjuku-ku, Tokyo, 160-8582 Japan

**Keywords:** Pancreaticoduodenectomy, Cholangitis, Late complication, Alkaline phosphatase

## Abstract

**Background:**

Because the survival rate for patients experiencing late complications after pancreaticoduodenectomy (PD) is increasing, late complications should receive as much attention as early complications do.

**Methods:**

Between April 2007 and August 2016, 133 patients underwent PD at our institution. We analyzed their cases to determine the predictors of late cholangitis after PD.

**Results:**

Of the 133 patients, 28 (21.1%) were diagnosed with postoperative cholangitis. A multivariate analysis showed that abnormal postoperative values of alkaline phosphatase were independently associated with postoperative cholangitis (odds ratio, 3.81; 95% confidence interval, 1.519–9.553; *P* = 0.004). The optimal cut-off value for postoperative alkaline phosphatase calculated from the receiver operating characteristic curve was 410 IU/L (sensitivity, 76.2%; specificity, 67.9%; area under the curve, 0.73). A univariate analysis to identify risk factors showed that pneumobilia was significantly related to a postoperative alkaline phosphatase value ≥ 410 IU/L (*P* = 0.041).

**Conclusion:**

This study suggests that an alkaline phosphatase level ≥ 410 IU/L is a predictor of late postoperative cholangitis. In addition, pneumobilia is also related to the postoperative alkaline phosphatase level. Therefore, alkaline phosphatase levels should be carefully monitored in patients with postoperative pneumobilia in the late postoperative course.

## Background

The survival rate for patients undergoing pancreaticoduodenectomy (PD) for peripancreatic carcinoma is increasing due to improvements in operative techniques, perioperative management, and early detection. Thus, late complications after PD should receive as much attention as early complications do. Reported early complications after PD include pancreatic fistula, delayed gastric emptying, infectious complications, and biliary complications [1, 2]. Several studies focused on late complications after PD have been reported. However, very few have focused on late biliary complications, especially postoperative cholangitis.

Most patients with late postoperative cholangitis are managed with conservative therapy. Because severe cholangitis is a critical condition, it is often difficult to manage. Moreover, severe and recurrent cholangitis may prevent recovery after surgery. However, the mechanism underlying postoperative cholangitis has not been clarified yet.

The aim of this retrospective study was to determine the predictors of late cholangitis after PD.

## Methods

Between April 2007 and August 2016, 133 patients underwent PD at our institution. Preoperative demographic and clinical data and details related to the surgical procedure and postoperative course were collected retrospectively. We analyzed these data to determine the predictors of cholangitis after PD. The Clinical Ethics Committee of our hospital approved this study, and informed consent was waived.

If patients were diagnosed with a biliary abnormality such as liver dysfunction, jaundice, cholangitis, and/or bile duct dilatation due to a periampullary tumor, preoperative biliary drainage was performed. The method of biliary drainage (i.e., endoscopic nasobiliary drainage [ENBD], endoscopic retrograde biliary drainage [ERBD], or percutaneous transhepatic biliary drainage [PTBD]) was chosen by a gastroenterologist in accordance with local policy.

All operations were performed by experienced pancreatic surgeons. Lymph nodes around the head of the pancreas, the common hepatic artery, and the hepatoduodenal ligament were dissected during pancreatectomy. After resection, reconstructions were developed according to the modified Child method or Traverso method. After resection, anastomoses were constructed to a single jejunal loop, which was brought through the transverse mesocolon in a retrocolic manner. First, a pancreaticojejunal anastomosis was performed in an end-to-side fashion. After pancreaticojejunal anastomosis, hepaticojejunostomy was performed with an end-to-side single layer of interrupted sutures using 4–0 absorbable suture materials. In general, we did not use biliary stenting. In cases of a small bile duct, an internal drainage tube was placed in the anastomosis. Finally, the operation was completed with an end-to-side duodenojejunostomy or gastrojejunostomy 40 cm downstream from the hepaticojejunostomy.

Perioperative management was standardized. All patients received broad-spectrum antibiotics for 1 day. No prophylactic somatostatin or octreotide was used. The nasogastric tube was removed on the first postoperative day when discharge was less than 500 mL. Total parenteral nutrition was used only in patients who could not tolerate a diet after postoperative day 5. Peripancreatic drains were removed if there was no evidence of leakage. If there was evidence of leakage or suspicion of infective complications (fever, leukocytosis, or purulent drain fluid), peripancreatic drains were left in situ, and a contrast-enhanced computed tomography (CT) scan was performed to determine if there was any intra-abdominal collection.

Patients underwent follow-up consisting of laboratory tests and ultrasonography or CT every 3 months during the first 3 years postoperatively. After 3 years, they were followed at 6-month intervals. Postoperative alkaline phosphatase concentrations greater than the normal range (104–338 IU/L) were regarded as abnormal. Patients who were followed with no evidence of cholangitis within 1 year after surgery were excluded to avoid future migrations.

Cholangitis was defined based on systemic inflammation, cholestasis, and imaging findings, in accordance with the updated Tokyo Guidelines (TG13) [3]. Our study included patients with a suspected diagnosis based on the TG13 diagnostic criteria for acute cholangitis. Symptoms occurring in the first month after surgery were excluded to avoid confusion caused by contamination due to an inflammatory response related to surgical stress. All the patients who were diagnosed as having cholangitis were hospitalized, and antibiotic treatments were started promptly.

Pancreatic fistula was defined according to the guidelines of the International Study Group on Pancreatic Fistula (ISGPF) [4]. Grades B and C were considered to be clinically relevant in this study. Bile leakage was defined according to the guidelines of the International Study Group of Liver Surgery (ISGLS) [5] as a drain bilirubin concentration of at least three times of the serum bilirubin concentration. Delayed gastric emptying was defined by the guidelines of the International Study Group of Pancreatic Surgery (ISGPS) [6]. Patients with all grades (grades A, B, or C) of delayed gastric emptying were enrolled in this study.

Continuous data are expressed as the mean ± standard deviation (SD). The chi-squared test or Fisher’s exact test was used to compare categorical data, and Student’s *t* test or the Mann-Whitney *U* test was used for continuous data, as appropriate. A logistic regression analysis was performed for a multivariate analysis to determine the independent risk factors. A *P* value < 0.05 was considered to be statistically significant. Analyses were performed using SPSS 19.0 software (SPSS Japan Inc., Tokyo, Japan).

## Results

One hundred and thirty-three consecutive patients underwent PD between April 2007 and August 2016, consisting of 77 men and 56 women with an average age of 67.2 years (range, 44–85). The average total bilirubin value of all patients before biliary drainage was 4.94 ± 5.49 mg/dL. Overall, 93 (69.9%) patients underwent preoperative biliary drainage. The diagnoses of the patients included pancreatic carcinoma (*n* = 49, 36.8%), cholangiocarcinoma (*n* = 49, 36.8%), ampullary carcinoma (*n* = 13, 9.8%), intraductal papillary mucinous neoplasm (*n* = 6, 4.5%), neuroendocrine tumor (*n* = 5, 3.8%), and others (*n* = 11, 8.3%). The types of operations performed were as follows: 20 (15.0%) PD, 41 (30.8%) subtotal stomach-preserving PD (SSPPD), and 72 (54.1%) pylorus-preserving PD (PPPD). The average operation time and blood loss for all patients were 454.3 ± 99.8 min and 990.6 ± 701.3 mL, respectively. Transfusions were performed in 33 (24.8%) patients. The characteristics of all patients are listed in Table 1.

**Table 1:** Characteristics of patients who underwent pancreaticoduodenectomy

Characteristic	*n* = 133
Age (years)^a^	67.2 ± 9.4
Sex	
Male	77
Female	56
Diagnosis	
Pancreatic carcinoma	49
Cholangiocarcinoma	49
Ampullary carcinoma	13
Intraductal papillary mucinous neoplasm	6
Neuroendocrine tumor	5
Others	11
Total bilirubin (mg/dL)^a^	4.9 ± 5.5
Preoperative biliary drainage	
Yes	93
No	40
Operation	
PD	20
SSPPD	41
PPPD	72
Operation time (min)^a^	454.3 ± 99.8
Blood loss (mL)^a^	990.6 ± 701.3
Transfusion	
Yes	33
No	100

*PD* pancreaticoduodenectomy, *SSPPD* subtotal stomach-preserving pancreaticoduodenectomy, *PPPD* pylorus-preserving pancreaticoduodenectomy

^a^Data are presented as mean ± standard deviation or *n* (%)

**Fig. 1:**
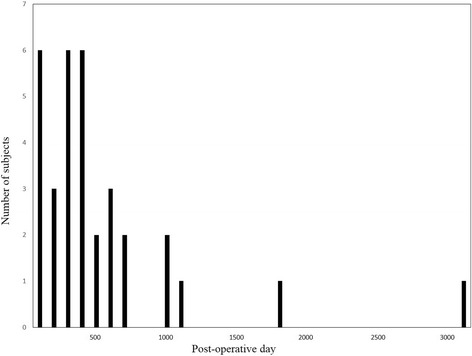
The time of postoperative cholangitis after surgery. Postoperative cholangitis occurred within the first year after pancreaticoduodenectomy in 15 patients (53.6%) and within 2 years after surgery in 23 patients (82.1%). Cholangitis occurred more than 1000 days postoperatively in the remaining 5 patients (17.9%)

Of the 133 patients, 28 (21.1%) were diagnosed with postoperative cholangitis. The median duration to postoperative cholangitis onset was 275 (range, 30–3037) days after surgery. The median follow-up duration was 861 (range, 74–3000) days after surgery. Postoperative cholangitis occurred within the first year after surgery in 15 patients (53.6%) and within 2 years after surgery in 23 patients (82.1%) (Fig. 1). Cholangitis occurred more than 1000 days postoperatively in the remaining 5 patients (17.9%). The frequency of postoperative cholangitis was 1.8 ± 1.3 (range, 1–5) times. Postoperative cholangitis occurred more than twice in 11 (39.3%) patients.

Table 2 shows the patient characteristics. The preoperative, perioperative, and postoperative characteristics were compared between the postoperative cholangitis group and no-cholangitis group. In the univariate analysis, there was no significant difference in age, sex, diagnosis, presence of diabetes mellitus, jaundice, preoperative biliary drainage, type of operation, operation time, blood loss, whether a transfusion was required, pancreatic fistula, bile leakage, delayed gastric emptying, or pneumobilia between the two groups. Significant differences were found in the rate of patients with a body mass index ≥ 24 kg/m^2^ (*P* = 0.039) and with an abnormal postoperative value of alkaline phosphatase (*P* = 0.003). The multivariate analysis showed that an abnormal postoperative value of alkaline phosphatase was independently associated with postoperative cholangitis (odds ratio, 3.81; 95% confidence interval, 1.519–9.553; *P* = 0.004).

**Table 2:** Analyses of risk factors for late postoperative cholangitis

		Univariate			Multivariate		
		Late postoperative cholangitis			Odds ratio	95% CI	*P* value
		(+)	(−)				
		*n* = 28	*n* = 105	*P* value			
Age (years)	≥ 70	11	46	0.667			
	< 70	17	59				
Sex	Male	17	60	0.734			
	Female	11	45				
Body mass index (kg/m^2^)	≥ 24	15	34	0.039	2.147	0.891–5.174	0.089
	< 24	13	71				
Diagnosis	Benign	1	8	0.684			
	Malignancy	27	97				
Diabetes mellitus	Yes	9	23	0.260			
	No	19	82				
Jaundice (≥ 2.0 mg/dL)	Yes	16	58	0.857			
	No	12	47				
Preoperative biliary drainage	Yes	16	77	0.097			
	No	12	28				
Type of operation	PD/SSPPD	9	52	0.101			
	PPPD	19	53				
Operation time (min)	≥ 420	16	64	0.715			
	< 420	12	41				
Blood loss (mL)	≥ 800	16	57	0.787			
	< 800	12	48				
Transfusion	Yes	7	26	0.979			
	No	21	79				
Pancreatic fistule grade B/C	Yes	6	13	0.224			
	No	22	92				
Bile leakage	Yes	1	1	0.378			
	No	27	104				
Delayed gastric emptying	Yes	0	9	0.203			
	No	28	96				
Surgical site infection	Yes	5	17	0.833			
	No	23	88				
Pneumobilia	Yes	15	41	0.167			
	No	13	64				
Alkaline phosphatase level^a^	Abnormal	20	42	0.003	3.81	1.519–9.553	0.004
	Normal	8	63				

*PD* pancreaticoduodenectomy, *SSPPD* subtotal stomach-preserving pancreaticoduodenectomy, *PPPD* pylorus-preserving pancreaticoduodenectomy

^a^Normal range of alkaline phosphatase level, 104–338 IU/L

**Fig. 2:**
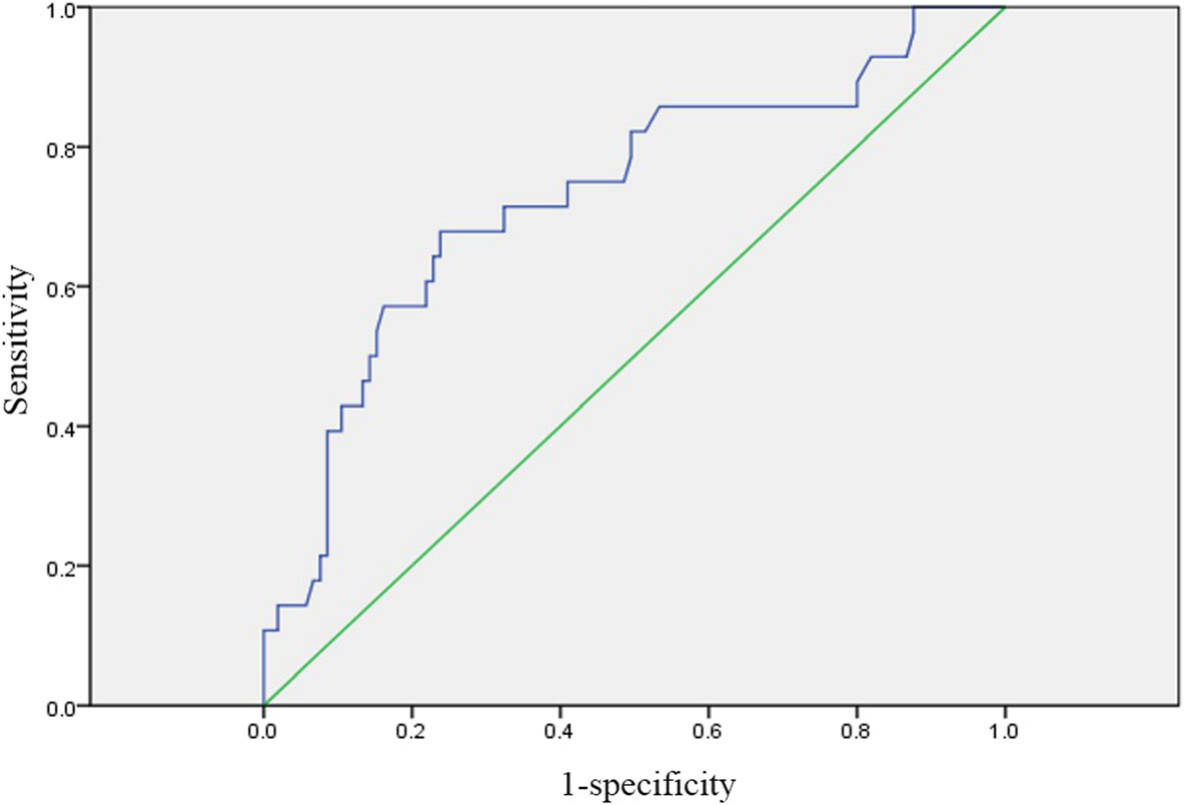
The ROC curve for alkaline phosphatase values and postoperative cholangitis in patients who underwent pancreaticoduodenectomy. The ROC curve was constructed to evaluate the optimal cut-off point for the postoperative value of alkaline phosphatase as a predictive factor associated with the incidence of postoperative cholangitis

Regarding the factors associated with the incidence of postoperative cholangitis, a receiver operating characteristic (ROC) curve was constructed to evaluate the optimal cut-off point for the postoperative value of alkaline phosphatase. The optimal value calculated by the ROC curve was 410 IU/L (sensitivity, 76.2%; specificity, 67.9%). The area under the curve (AUC) was 0.73 (Fig. 2).

A univariate analysis was performed to identify risk factors for the presence of a postoperative alkaline phosphatase value ≥ 410 IU/L. Pneumobilia was significantly related to a postoperative alkaline phosphatase value ≥ 410 IU/L (*P* = 0.041) (Table 3).

**Table 3:** Relationship between alkaline phosphatase value and other factors

		Alkaline phosphatase		
		≥ 410 IU/L	< 410 IU/L	
		*n* = 44	*n* = 89	*P* value
Age (years)	≥ 70	22	35	0.242
	< 70	22	54	
Sex	Male	27	50	0.569
	Female	17	39	
Body mass index (kg/m^2^)	≥ 24	17	32	0.763
	< 24	27	57	
Diagnosis	Benign	2	7	0.717
	Malignancy	42	82	
Diabetes mellitus	Yes	13	19	0.298
	No	31	70	
Jaundice (≥ 2.0 mg/dL)	Yes	26	48	0.573
	No	18	41	
Preoperative biliary drainage	Yes	32	61	0.620
	No	12	28	
Type of operation	PD/SSPPD	20	41	0.947
	PPPD	24	48	
Operation time (min)	≥ 420	31	49	0.088
	< 420	13	40	
Blood loss (mL)	≥ 800	29	44	0.073
	< 800	15	45	
Transfusion	Yes	13	20	0.374
	No	31	69	
Pancreatic fistule grade B/C	Yes	8	11	0.367
	No	36	78	
Bile leakage	Yes	2	0	0.108
	No	42	89	
Delayed gastric emptying	Yes	2	7	0.717
	No	42	82	
Pneumobilia	Yes	24	32	0.041
	No	20	57	

*PD* pancreaticoduodenectomy, *SSPPD* subtotal stomach-preserving pancreaticoduodenectomy, *PPPD* pylorus-preserving pancreaticoduodenectomy

## Discussion

Postoperative cholangitis is likely to occur when the sphincter of Oddi is resected with a barrier function of reflux. As an early complication, the incidence of postoperative cholangitis was 1.0–16.6% of patients [7–10]. However, it might be difficult to diagnose cholangitis accurately in the early postoperative course because of contamination from an inflammatory response related to surgical stress. On the other hand, late biliary complications, such as postoperative cholangitis and biliary stricture, have been reported. Of these, postoperative cholangitis is a rarely encountered complication that may require emergent hospital readmission [11]. The reported long-term outcomes of biliary-enteric anastomoses were as follows: the incidence of postoperative cholangitis was 10.9% in the choledochoduodenostomy group and 6.4% in the hepaticojejunostomy group [12]. Late cholangitis after PD has reportedly occurred in 6.7–14.4% [7, 13, 14] of patients. These results are slightly lower than our result that 28 (21.1%) of 133 patients were diagnosed with postoperative cholangitis.

It is well known that postoperative cholangitis is caused by a biliary obstruction, such as biliary stricture, bile stasis, or stone. In addition, other reasons for postoperative cholangitis are as follows: intestinal obstruction, afferent limb syndrome, and stasis due to jejunal peristaltic failure [15]. Biliary stricture, defined as the need for endoscopic, percutaneous, or surgical intervention [14, 16], may cause postoperative cholangitis following bile stasis. Bile stasis is considered to be associated with bacterial growth in the bile juice [17]. Duconseil et al. found that 47% of 17 patients with biliary stricture developed postoperative cholangitis [16]. In our study, 2 patients with biliary stricture were also diagnosed with cholangitis. In addition, 7 (25%) patients with postoperative cholangitis were found to have biliary dilatation and anastomotic stenosis. However, follow-up CT evaluation revealed that there was no evidence of biliary obstruction after conservative therapy. Parra-Membrives et al. found even when postoperative cholangitis is caused by a true biliary stricture, about 40% of patients have a recurrent episode without a proven biliary stricture [14]. Thus, it was suggested that they had retained activity in the biliary tree. Therefore, there is a limitation to determining biliary obstruction without cholangitis by using CT. It seems reasonable to suppose that detecting bile stasis due to anastomotic stenosis before cholangitis is useful. In this study, as a predictive aid, results of the multivariate analysis showed that an abnormal postoperative value of alkaline phosphatase was independently associated with postoperative cholangitis. Additionally, pneumobilia was significantly related to a postoperative alkaline phosphatase value ≥ 410 IU/L. Another factor that causes postoperative cholangitis is bile stasis due to afferent limb syndrome; 50% of patients with afferent limb syndrome were reported to present with obstructive jaundice or cholangitis [15]. Despite bile stasis, proven afferent limb obstruction may be detected, because it was considered to be responsible for the reconstruction method chosen [18]. The duration to cholangitis onset after PD was fascinating. In this study, the median duration to postoperative cholangitis onset was 275 (range, 30–3037) days after surgery, and postoperative cholangitis occurred at a rate of approximately 50% within a year and of 80% within 2 years, respectively. Our results for the duration to cholangitis onset were similar to those previously reported for biliary stricture. The median reported duration to biliary stricture after PD was reported to be 13 months (range, 1 month to 9 years) [19] and 205 days (range, 12–1380 days) [16], respectively. Hence, it was assumed that postoperative cholangitis was associated with biliary stricture.

A previous study showed that preoperative biliary drainage with surgery for cancer of the head of the pancreas significantly increased the rate of postoperative cholangitis [20]. On the other hand, another study showed that preoperative biliary drainage was not associated with postoperative cholangitis. However, it has been suggested that the incidence of postoperative cholangitis was significantly higher in patients with bile duct carcinoma and was significantly associated with hospitalization and intensive care unit stay [13]. The efficacy of preoperative biliary drainage for postoperative cholangitis remains controversial. Few studies have reported on late postoperative cholangitis. In our investigation of postoperative cholangitis, the multivariate analysis showed that an abnormal postoperative value of alkaline phosphatase was independently associated with postoperative cholangitis. Additionally, we focused on the impact of the alkaline phosphatase cut-off value demonstrated by the ROC curve, which we expect to be a predictor of postoperative cholangitis. Moreover, pneumobilia was significantly related to a postoperative alkaline phosphatase value ≥ 410 IU/L. Chan et al. reported that pneumobilia was detected in 52% of patients with recurrent pyogenic cholangitis [21]. We compared differences between the postoperative cholangitis group and no-cholangitis group in this study, and there was not a significant difference in pneumobilia between the two groups. However, pneumobilia was significantly related to a postoperative alkaline phosphatase value ≥ 410 IU/L. Therefore, a strict follow-up that includes measurement of alkaline phosphatase levels should be provided, especially to patients with pneumobilia during the postoperative course.

Generally speaking, with the exception of postoperative day 1, patients received no antimicrobial therapy because unnecessary antimicrobial therapy could induce drug-resident bacteria. Cammann et al. reported that intraoperative bile culture as a prophylaxis for postoperative cholangitis was useful because it can be altered by antimicrobial prophylaxis [13]. Another study reported that a short course of postoperative antimicrobial therapy reduced the occurrence of infectious complications after PD [22]. Therefore, antimicrobial therapy was provided to patients at the time that cholangitis was diagnosed because little has been reported on antimicrobial prophylaxis for postoperative cholangitis. Both of the previous studies investigated complications in the early postoperative course. Prophylactic antibiotics for late postoperative cholangitis is still incompletely understood. In this study, 11 (39.3%) of 28 patients with postoperative cholangitis experienced recurrent cholangitis. Long-term exposure to bile juice due to biliary stasis, reflux, or infection may arise from cholangiocarcinoma [23]. An experimental study demonstrated that exposure to digestive enzymes and bacteria caused epithelial hyperplasia in rats [24]. Moreover, Tocchi et al. reported the long-term outcomes for patients undergoing biliary-enteric anastomosis [12]. The incidence of cholangiocarcinoma was 7.6% after choledochoduodenostomy and 1.9% after hepaticojejunostomy. Reflux of digestive fluid and bacteria by recurrent cholangitis is considered a risk factor for carcinogenesis of the choledochal epithelium. Although most patients with postoperative cholangitis improve with conservative therapy, patients with frequent recurrence should be considered for improvement measures.

As noted, the mechanism underlying postoperative cholangitis is still unclear. How to prevent these complications should be considered. Few studies have focused on preoperative biliary drainage. A previous report showed that preoperative biliary drainage was associated with a rate of complications that was significantly higher than that in the early-surgery group [20]. In particular, the biliary drainage group had a higher incidence of postoperative cholangitis (26%) than did the early-surgery group (2%). On the other hand, Sahora et al. reported that there was no significant difference in the overall postoperative morbidity and mortality between the two groups [25]. However, the number of positive bile cultures was significantly higher in the preoperative biliary drainage group than in the non-preoperative biliary drainage group. Thus, taken together, the results obtained so far are controversial. In addition, regarding postoperative biliary drainage, the incidence of postoperative cholangitis was significantly higher in patients with external stents (25%) than in patients with no stents (3.8%). The limitation of this study was the small number of patients and its retrospective nature. Furthermore, Hiyoshi et al. reported that hepaticoplasty to widen the small bile duct was useful for preventing postoperative cholangitis [26]. Since late postoperative cholangitis may occur suddenly, it would be helpful to predict its occurrence.

Our study has several limitations. First, a small number of patients were included to investigate postoperative cholangitis. Second, it is occasionally difficult to diagnose cholangitis after PD, so there may have been bias because of contamination of postoperative conditions. Third, the follow-up duration for patients without cholangitis was not sufficient to exclude its occurrence entirely. This is supported by our result showing that approximately 20% of patients with postoperative cholangitis developed it more than 2 years after PD. Thus, patients who might have developed postoperative cholangitis in the future may have been included in the group of patients without postoperative cholangitis. The final limitation of this study is the inability to assess for antibioprophylaxis and management of postoperative cholangitis. Therefore, a randomized controlled trial is required to confirm a specific postoperative management.

## Conclusion

A postoperative alkaline phosphatase value ≥ 410 IU/L was useful for predicting the development of late postoperative cholangitis. Additionally, pneumobilia was related to the postoperative alkaline phosphatase value. Therefore, careful follow-up is needed in the late postoperative course.

## Abbreviations

AUC: Area under the curve
ISGPF: International Study Group on Pancreatic Fistula
PD: Pancreaticoduodenectomy
PPPD: Pylorus-preserving pancreaticoduodenectomy
ROC: Receiver operating characteristic
SSPPD: Subtotal stomach-preserving pancreaticoduodenectomy
TG13: Tokyo Guidelines
